# Modifying quantitative sensory testing to investigate tactile sensory function and behavioral reactivity in children with intellectual and developmental disabilities: establishing feasibility and testing sex, autism, and self-injury effects

**DOI:** 10.1186/s11689-025-09603-x

**Published:** 2025-03-27

**Authors:** Jaclyn Gunderson, Emma Worthley, Breanne Byiers, Alyssa Merbler, Andrea Huebner, Deanna Hofschulte, Jasmine Lee, Catherine Riodique, Frank Symons

**Affiliations:** 1 Department of Psychiatry and Psychology, Mayo Clinic, 200 1st street SW, Rochester, MN 55905 USA; 2 College of Education and Human Development, University of Minnesota, Burton Hall, 178 Pillsbury Dr SE, Minneapolis, MN 55455 USA

**Keywords:** Sensory responsivity, Tactile reactivity, Intellectual developmental disability, Autism, Modified quantitative sensory test, Self-injurious behavior

## Abstract

**Background:**

Sensory reactivity differences are common across neurodevelopmental disorders (NDDs), however very few studies specifically examine tactile or pain responses in children with NNDs, especially those with communication challenges. The current study aimed to (a) replicate the feasibility of a modified quantitative sensory test (mQST) with a sample of children with NDDs, (b) assess validity evidence based on behavioral reactivity during mQST application and the corresponding behavioral measurement coding system, and (c) explore group differences in behavioral reactivity to mQST stimuli by demographic (sex), clinical (autism status), and behavioral pathology (self-injury) variables.

**Methods:**

The mQST protocol was implemented and blindly coded across 47 participants aged 2–12 years (*M* age = 6.7 years, *SD* = 2.6; 70% male) with NDDs. Feasibility was measured by completion of the mQST protocol and interobserver agreement. Validity was assessed using paired *t*-tests investigating differences between behavioral reactivity to active stimuli compared to a sham trial. Boxplots were used to visually explore differences in group characteristics (sex, autism status, and self-injurious behavior), with two-sample *t*-tests used to further characterize differences in SIB group characteristics in behavioral reactivity to mQST stimuli.

**Results:**

The mQST provided codable data across 91% of stimuli applications with high IOA (84.7% [76.7–95%]). Behavioral reactivity was significantly higher for active vs. sham stimuli. Children reported to engage in self-injurious behavior showed significantly more reactivity to the second half of the repeated von Frey stimulus application compared to children without caregiver-reported self-injurious behavior (M = 6.14, SD = 3.44), t (40)= -2.247, *p* =.04).

**Conclusion:**

The mQST is a feasible approach to investigate tactile reactivity in children with NDDs and complex communication needs. The mQST may be useful in understanding sensory variables in relation to developmental and behavioral outcomes such as self-injurious behavior.

There are longstanding health and behavior outcome concerns regarding sensory function and dysfunction among individuals with neurodevelopmental disorders (NDDs), including intellectual disabilities (ID) and autism spectrum disorder (ASD) [1]. The historical narrative of sensory functioning in general and tactile function (touch, pain) in particular was the belief that individuals with NDDs were insensitive or indifferent to sensations such as pain; indeed such beliefs were the basis for pain and touch reactions being part of early IQ tests (see Sobsey, 2006 for an in depth historical review) [2]. Elements of this perspective extended into models of pain concerning self-injurious behavior (SIB) such that it was assumed that if SIB was present, it was likely that pain regulatory systems were aberrant, most likely in the direction of being blunted [3]. The dominant assumption regarding pain and its function in NDDs has more recently given way to emerging evidence that individuals with NDDs do experience and express pain and pain behavior [4, 5]. In a review article, Moore [6] found that, although evidence based on proxy and self-reported studies suggested that individuals with ASD were hyposensitive to pain, studies based on direct observation and including experimental pain assessments showed normal or hypersensitive responses to pain.

More recently, studies have shown increased facial reactivity to experimental tactile stimulation among adults with mild to moderate ID relative to controls [7]. These studies suggest important differences between perceived and objectively measured tactile and pain responses among individuals with NDDs, although it is unclear whether the patterns are different among individuals with ASD versus those with other NDDs. Additionally, most of the extant work has been conducted among adults with mild to moderate disabilities, due to difficulties in conducting experimental pain assessments among individuals with communication challenges, including young children and those with more severe ID.

Quantitative sensory testing (QST) is a common psychophysics-based method used to assess sensory threshold functioning corresponding to different peripheral sensory afferent fiber types and their properties and central nervous system regulatory pathways by applying modality specific tactile stimuli (e.g., heat, cold, sharp edge) onto the skin [8, 9]. QST methods are routinely used to screen for and diagnose peripheral and central nervous system diseases and used in clinical research investigating underlying pathological mechanisms of chronic pain disorders, particularly in neuropathic pain [10–12]. 

There are two main approaches used in the application of QST. One QST approach utilizes the method of limits to determine somatosensory thresholds using stimuli (e.g., thermal heat) that are applied with repeated intensity until the subject indicates by verbally responding or pressing a button (see Yarnitsky & Sprecher, 1994) [13]. The method of limits is dependent on reaction time (e.g., verbal report or pressing a button) and ability to follow directions (i.e., press the button or say “pain” when the stimulus becomes painful), that invalidate the use of such testing procedures with populations that cannot self-report, may not be able to follow directions, or have motor delay or dysfunction. The other dominant QST approach uses the method of levels in which calibrated stimuli of the same intensity are used with all participants. For the method of levels, at the end of each stimulus presentation, the individual is asked whether the stimulus was perceived. The response– yes/no - determines the next level of stimulus intensity (higher or lower). In either approach– if or when self-report (or understanding) is compromised– the valid application of QST is jeopardized.

Considering the communicative, motor, and cognitive impairments often associated with NNDs, one strategy to improve our understanding of somatosensory functioning in individuals has been to rely on nonverbal behavioral indicators of pain expression (e.g., gross motor behavior, facial expression, mood/affect ratings, etc.). Previous research demonstrates that individuals with NDDs who cannot reliably self-report are capable of displaying a behavioral response associated with the timing of a painful stimulus [14–16]. Based on this approach, Symons and colleagues [17] modified QST (mQST) to quantify nonverbal behavioral reactivity to time-locked applications of conventional QST stimuli but without dynamic ramps of stimulus intensity, so thresholds, per se, are not established. The loss of established threshold was offset, perhaps, by the calibrated application of stimulus types with known quantitative intensities.

The mQST approach has been applied to various developmental disability groups since it was first described by Symons et al. [17]. Barney and colleagues [18] evaluated the feasibility of the mQST approach with 20 young children with global developmental delay (GDD) and compared behavioral response profiles to children without developmental delay. Children with GDD exhibited observable and quantifiable behavioral reactions (i.e., facial, gross motor, and vocal) to mQST stimuli. Children with GDD, on average, were significantly more reactive to some stimuli compared to others, particularly the repeated von Frey stimulus (an industry standard tactile device with a thin elastic nylon fiber that can be calibrated on intensity by fiber thickness, applied directly on the skin) when compared to all other stimuli except for the pin prick. For our purposes, the study provided initial feasibility evidence of the mQST for use with samples of children with GDD. The mQST was also successfully implemented with a Rett syndrome sample in a study that reported resting heart rate variability predicted behavioral reactivity to mechanical stimulation [19]. Additionally, the mQST was used to profile sensory function by pain outcome group for individuals with CP following intrathecal baclofen implant surgery [20]. The mQST has also been used to objectively measure behavioral reactivity in a sample of children with GDD in relation to peripheral innervation biomarkers [21]. The subgroup of children with high behavioral reactivity during the mQST had significantly reduced epidermal nerve fiber densities as compared to the group with average behavioral reactivity, indicating that the mQST provided an approach to interrogate tactile and acute nociceptive reactivity associated with a peripheral biomarker relevant for sensory and pain processing for some children. In an adult study, researchers using the mQST found that individuals with chronic SIB had higher rates of behavioral reactivity compared to those without SIB [17]. It is plausible that tactile and nociceptive sensory response profiles exist in individuals with NDDs and may help delineate functional differences in sensory mechanisms that could ultimately relate to the pathophysiology underlying chronic SIB. Considering sensory differences are a key characteristic of ASD [20] with evidence suggesting tactile sensitivity in up to 60% of autistic individuals [21, 22] and high rates of SIB within the autistic population [23, 24], it is important to examine behavioral reactivity across ASD status. Furthermore, there is mixed evidence of potential sex differences in tactile and nociceptive responsiveness. Some research suggests greater reactivity in females [25–27], while other research suggests no difference by sex [28, 29]. Overall, this line of research has been dominated by caregiver report assessment with little direct testing research available.

The objective of the current study was to further investigate the feasibility, validity, and utility of the use of the mQST protocol to quantify behavioral reactivity to calibrated tactile stimuli in a pediatric sample with NDD. Specifically, the current study aimed to (a) replicate the feasibility of the mQST approach with a sample of children with NDD (establishing whether children can complete the protocol and that the protocol can be reliably implemented), (b) assess validity evidence that the mQST approach and corresponding behavioral measurement system was specific to behavioral reactivity during tactile stimuli application (there are differences in behavioral responses measured during stimulus application compared to sham trials), and (c) explore whether behavioral reactivity to mQST and its different stimuli differed by demographic (sex), clinical (ASD status), or behavioral pathology (SIB) variables.

## Method

### Participants

This preliminary analysis presents data from 47 participants (*M* age = 6.6 years, *SD* = 2.6; 70% male) with behaviorally coded mQSTs from a broader sample of 149 participants enrolled in a larger study protocol. Participation in the full protocol included annual survey questions related to restricted and repetitive behavior and self-selected involvement in research activities, including biomarker procurement (saliva, blood, skin biopsy), mQST, and telehealth-supported home visits. The working sample of *n* = 47 participants was created by selecting all children from a larger sample with fully coded mQST sessions. Participants were recruited from a specialty neurodevelopmental diagnostic clinic at a medical center. Study inclusion criteria were: (a) developmental delay defined by intellectual disability or delays in at least two domains of development documented in the medical record, and (b) parent/guardian consent for participation. Study exclusion criteria included serious accompanying health problems associated with terminal (e.g., lysosomal storage diseases) or neurodegenerative diseases (e.g., muscular dystrophy). ASD diagnosis was collected retrospectively from medical records for sample description purposes only. All participants received a multidisciplinary autism spectrum disorder evaluation. The multidisciplinary team included a developmental behavioral pediatrician, speech language pathologist, audiologist, social worker, nurse, and neuropsychologist. Evaluations included standardized testing such as the Autism Diagnostic Observation Schedule, Second Edition (ADOS-2) [30] and/or the Childhood Autism Rating Scale, Second Edition (CARS-2) [31]. No additional diagnostic procedures were completed for the current study. The study was approved by the Mayo Clinic and MHealth Fairview IRB, and all participants gave written informed consent.

### Procedures

#### Modified quantitative sensory test (mQST)

##### Setting

To complete the mQST, the participant and at least one caregiver were directed to a quiet testing room at the medical center. The child sat on a chair with footrests so that their lower legs were accessible to the examiner seated on the floor to the child’s left. Stimuli were placed outside the child’s view and covered by a cloth blanket. A tray was placed across the chair’s lap to keep the stimuli and examiner’s approach from the child’s view. Caregivers remained with their child during the sensory tests and provided the option of using a sticker chart to visually guide the child through the exam with a sticker placed on the chart after each stimulus. Two video cameras captured the stimuli application and child behavior for later behavioral coding. One digital video camera was positioned on a tripod approximately 2.5 m from the participant with a viewing frame including the participant’s whole body and face. A second camera was held by a research team member about one meter away to collect a close-up view of the child’s face.

##### Stimuli

The mQST was conducted using a protocol similar to previous investigations [32, 33]. We collected a 30-second baseline video recording of the child in the examination chair for the child to acclimate to the environment and for behavioral coders to identify baseline behaviors not related to stimulus application. The stimuli consisted of six calibrated stimuli and two sham trials in the same order, except for the randomized sham. An initial sham trial was conducted for every mQST, where a von Frey monofilament with the fiber closed was held approximately two inches away from the calf, consistent with all other applications except for the actual touch. Light touch was applied once per second for 5 s by hand with a von Frey monofilament (2.0 g) pressed against the skin until the filament bent at a 45° angle. A light pin prick was applied for less than one second with a plastic neurological exam pin (Medipin; US Neurologicals). Cold touch was applied lightly for five seconds using a room temperature metal thermal probe (approximately 22 °C; Tip Therm, US Neurologicals). Deep pressure was applied by hand using an algometer (Wagner Instruments) pressed into the calf at a consistent 4 lbs of pressure for 5 s. A repeated von Frey monofilament (60 g) was applied by hand at approximately 1 Hz for 30 s. Heat was applied for five seconds using a 3-mm electronic thermal heat probe (WR Medical Electronics) at a temperature of 50 C. The randomized sham was conducted in the same way as the initial sham but was randomized into the sequence.

Stimuli were applied to the back of the child’s bare left and then the right calf. Each stimulus approach and removal were audibly signaled and accompanied by a 5-second lag between signal and application. Timing was guided by an in-ear digital timer. At least a ten-second return to baseline for child behavior separated the application of each stimuli type. If a participant had tears or other signs of distress, the stimulus application was terminated. If distress was ongoing after stopping the application, the entire mQST was terminated at the lead examiner and/or caregiver’s discretion.

##### Behavioral coding

Behavioral reactivity was scored from video recordings using a modified Face, Legs, Activity, Cry, Consolability (FLACC) observational coding system [34]. Coders were blinded to the stimuli type being applied and participant diagnostic information. Reactivity was scored across five behavior classes: upper face (e.g., brow furrow, eye squeezes), lower face (e.g., grimace, mouth open), limb being touched (e.g., leg flinch, move the leg away), activity of whole body (e.g., tensing, movements of other limbs), and vocalizations (e.g., “that hurts,” “that’s cold,” whine). Each behavior class was coded from 0 to 2 for each stimulus application, considering the frequency, duration, and intensity of the defined behaviors to determine a 1 vs. 2 score. For example, a short or small brow furrow would be scored as a 1 for the upper face, but an intense or negative furrow or a furrow lasting more than half of the application time would be scored a 2. Scoring intensity (i.e. 1 vs. 2) also considered each participant’s gross motor ability to ensure those with more limited gross motor function could still potentially reach a score of 2 (more intense reactivity). Only defined behaviors that started during application or changed from the participant’s baseline level during application were scored. Behavioral reactivity for each stimulus type on each calf could range from 0 to10. From the mQST coding data, scores from all five behavior classes were summed to give a total score for each calf for each stimulus. Then, we averaged the scores across both calves for each stimulus to derive stimuli-specific reactivity scores for each participant. Terminated stimuli were scored with the maximum behavioral reactivity score of 10. Stimuli not applied due to termination of the rest of the test were not given a score. To account for the longer application length of the repeated von Frey (30 s) compared to all other stimuli (5 s), the repeated von Frey was behaviorally scored for the first 5 s of application and the last 5 s of application, resulting in two scores for this stimulus. Behavior coding staff were trained to a 90% criterion with the lead coder using exact agreement calculations at the level of each behavior category for each stimulus application ([# exact score agreements/# total scored behavior categories] x 100). 17% of videos (*n* = 8) were used for training and/or were consensus-coded due to difficult baseline determinations. For training and consensus-coded videos, agreed-upon baselines were set, then videos were independently coded by the lead and a secondary coder, and then disagreements were consensus-coded (mean pre-consensus IOA with the lead coder = 78.2%). IOA was completed on 46% of the remaining independently coded videos.

### Questionnaires

The RBS-EC is a 34-item caregiver-rated measure covering 4 subscales: (a) Repetitive Motor, (b) Ritual and Routine, (c) Restricted Interests and Behavior, and (d) Self-directed Behavior. Caregivers rate how often a behavior occurs based on a 5-level rating scale (0 = *behavior does not occur* to 4 = *behavior occurs many times a day*). The extent to which each category of behavior interferes with other activities or interactions is also judged on a 4-level rating scale (0 = *never* to 4 = *always*). Total endorsed scores from the 7-item Self-Directed subscale were used to measure the presence of SIB, including questions about frequency of self-directed hitting, biting, rubbing, scratching, poking, pinching, pulling own hair, and skin picking. For the purposes of this study, the SIB group was defined by caregiver-reported presence of self-injurious behavior with a score of 1 or greater on any self-directed subscale item. The RBS-EC showed evidence of good overall internal consistency (α = 0.90) and strong test-retest reliability (ICC = 0.87 for topographies and 0.90 for frequency) [35]. Additional, validity evidence supports the use of the RBS-EC in children up to 7 years of age [36]. However, the RBS-EC was designed based on the Repetitive Behavior Scale–Revised (RBS-R) [37] and the self-directed items are the same as those from the RBS-R SIB subscale, which was created to measure SIB in adults with ID. Specifically, the items associated with the SIB subscale have been consistent across several analyses with populations from infancy to adulthood [35, 38–40] suggesting that the construct of SIB is relatively invariant across ages.

### Analysis

Participant characteristics were described in terms of race, ethnicity, ASD diagnostic status (i.e., ASD vs. developmental delays/disabilities [DD] without an ASD diagnosis), and interference of SIB. Descriptive statistics (i.e., mean and standard deviation) were computed for SIB variables from the RBS-EC, including the total score on the RBS-EC self-directed behavior subscale, and the mQST stimuli-specific behavioral reactivity scores.To pursue the first aim and assess the feasibility of the mQST approach in our sample, we calculated the percentage of completed stimulus applications across stimuli and the percentage of applications that were not codable (i.e., applications with missing camera footage or poor visibility). Additionally, we investigated if the mQST behavioral coding approach produced reliable scoring of behavioral reactivity to mQST stimuli by analyzing interobserver agreement (IOA; >80% agreement considered reliable). IOA was calculated using [# exact score agreements/# total scored behavior categories] x 100).

For the validity analysis in the second aim, paired *t*-tests were utilized to calculate differences between the initial sham stimulus and active mQST stimuli (e.g., light touch, pin, pressure, repeated Von Frey, heat). Effect sizes of differences between the initial sham and the active mQST stimuli were estimated using Cohen’s *d.* Multiple comparisons were controlled for using the Benjamini-Hochberg method.

The third aim was to explore potential group differences in behavioral reactivity to mQST stimuli by sex, ASD status, or SIB. Group differences were explored visually using box and whisker plots. Additionally, group differences between participants with and without caregiver-reported SIB were tested using two-sample t-tests for each mQST stimulus. We aggregated the mQST reactivity scores across individuals by SIB status as reported by the caregiver on the RBS-EC and investigated group differences in behavioral reactivity across mQST stimuli using two-sample *t*-tests. To investigate if there was information in terminated tests, group differences between the SIB group and the no SIB group were computed in addition to a description of the subgroup of participants who terminated the protocol. Differences between the SIB and no SIB group on meeting test termination criteria were computed using the Fisher’s exact test. This subgroup was described by the age range and percentages of assigned sex at birth, ASD diagnosis, and endorsed SIB.

To assess whether the mQST was useful to characterize differences in sensory reactivity for participants with and without SIB, we explored group differences using z-transformed mQST reactivity scores against the group of participants who did not self-injure and plotted the standardized group averages. The mQST behavioral reactivity scores were standardized by Z-transforming the reactivity scores against the group of individuals who did not self-injure (individuals with 0 endorsed SIB on the RBC-EC). This allowed us to aggregate the coded behavior for each stimulus trial and compare reactivity with individuals with no SIB. Z-scores were calculated for each mQST stimulus reactivity score based on methods described by Barney et al. [33] and the German Research Network on Neuropathic Pain [9] utilizing the following equation: Z-score = (X_i - mean_no SIB group) / SD_no SIB group. The Z score indicates how far above or below the mean of the (no SIB group) an individual’s score is and is a common transformation used in QST studies. The Z-transformed stimulus reactivity scores were plotted as averages for the SIB group across mQST stimuli to identify a sensory reactivity profile for the group of participants who self-injured (individuals with 1 or more endorsed self-injurious behaviors on the RBS-EC).

## Results

### Participants

A total of *n* = 47 participants (*M* age = 6.6 years, *SD* = 2.6; 70% male) were included in the data analysis for this study. Sample characteristics, including race, ethnicity, diagnostic category (ASD vs. DD), presence of SIB, and the extent to which SIB interferes in other activities, are presented in Table 1. The mean self-directed subscale score on the RBS-EC across the participants was 3.89 (SD = 4.44) and ranged from 0 to 16. Based on the proxy report RBS-EC, 77% (*n* = 36) of participants engaged in SIB that occurred at least weekly within the month before testing (self-directed subscale score ≥ 1) 13% (*n* = 6) indicated SIB behaviors interfered often or always. One participant was missing the item response related to how often SIB behaviors interfered.

**Table 1:** Participant characteristics

Characteristic	Total (*n* = 47)
Race	
Asian	1 (2.13%)
Asian/Indian	2 (4.26%)
Black/African American	3 (6.38%)
Other	5 (10.64%)
White	36 (76.60%)
Ethnicity	
Chose not to disclose	1 (2.13%)
Hispanic or Latino	1 (2.13%)
Not Hispanic or Latino	45 (95.74%)
Diagnosis	
ASD	34 (72.34%)
DD	13 (27.65%)
SIB-interfere	
Never	13 (27.66%)
Rarely	15 (31.91%)
Sometimes	12 (25.53%)
Often	4 (8.51%)
Always	2 (4.26%)

Note. ASD = autism spectrum disorder; DD = developmental delay/disorder without ASD; SIB = caregiver-reported presence of self-injurious behavior (≥ 1 RBS-EC self-directed behavior subscale; SIB-interfere = caregiver-reported self-injurious behavior as interfering on RBS-EC subscale

### Feasibility evidence

**Fig. 1:**
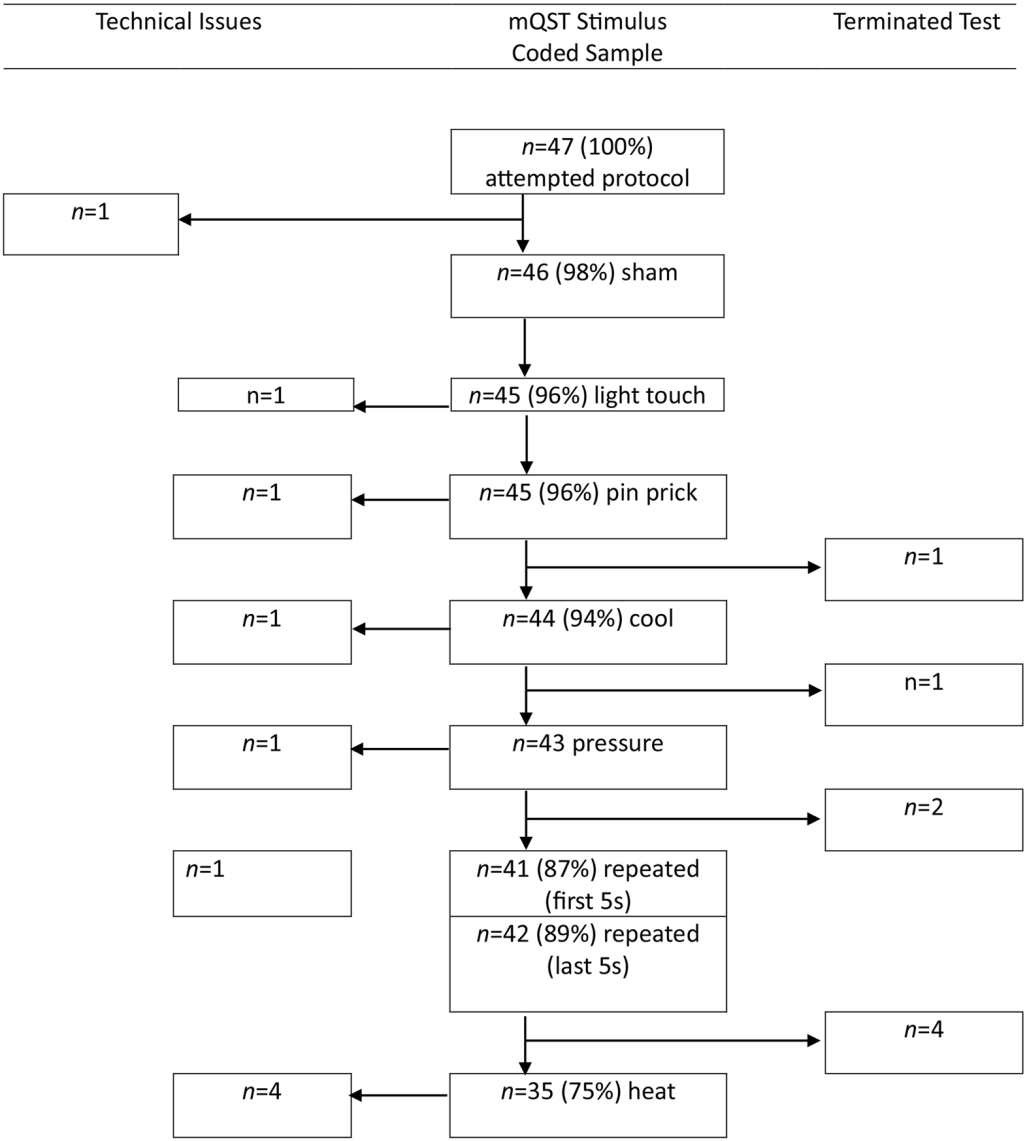
Missingness Reasons for mQST stimuli responsivity scores

The first aim of this study was to investigate the feasibility of the mQST approach in a sample of children with NDDs. Feasibility was assessed in part by participant completion of the mQST protocol and in part by the reliability of applying the behavioral reactivity coding scheme to the videos of participants. Figure 1 depicts participant completion and the flow of codable stimuli throughout the application of the mQST protocol. For one participant (2% of the sample), data were unusable for the entire protocol due to technical camera issues. Out of the 46 participants with mostly codable videos, 0 were missing data for sham, 1 participant (2.2%) was missing data for light touch, 1 (2.2%) was missing data for pin, 2 (4.3%) were missing data for cool touch, 3 (6.5%) were missing data for pressure, 5 (10.9%) were missing data for the first 5 s of repeated Von Frey, 4 (8.7%) were missing data for the last five seconds for repeated Von Frey, and 11 (23.9%) were missing data for heat (primarily due to the test being terminated prior). Of the reasons data was missing or unusable, 74% were due to termination (stopping) of the stimuli or protocol. In the case of the protocol being stopped, subsequent stimuli were not applied or scored. Other reasons for missing data included technical camera issues (18.5%), and the child’s face not being visible due to hands or hair covering the face (7.5%). Independent raters scored behavior across 45% of the mQST observations resulting in high IOA (mean = 84.7%, *SD* = 5.1, range = 76.7 − 95.0%).

### Validity evidence

To assess the validity of the mQST approach to measure behavioral reactivity to tactile stimuli for children with NDDs, we examined reactivity to tactile stimuli compared to an initial sham trial using paired *t*-tests. On average, for all participants with NDDs, behavioral reactivity was significantly higher for all stimuli compared to the initial sham with medium to large effect sizes (see Table 2). Across all children in the sample, behavioral reactivity was highest for the repeated von Frey last five seconds (*M* = 5.49, *SD* = 3.54), followed by the first five seconds (*M* = 4.59, *SD* = 2.59), deep pressure (*M* = 3.71, *SD* = 2.50), then pin (*M* = 3.70, *SD* = 2.24).

**Table 2:** Paired t-tests examining differences between mQST Sham and active stimuli

mQST Stimulus	M	SD	t	df	*p*	Cohen’s d
Sham	1.71	1.24				
Light touch	2.73	1.94	3.063	44	0.004	0.632
Pin	3.70	2.24	4.877	44	< 0.001	1.021
Cool	3.17	2.36	3.573	43	0.002	0.781
Pressure	3.71	2.50	4.935	42	< 0.001	1.027
Repeated Von Frey (first 5s)	4.59	2.59	6.668	40	< 0.001	1.444
Repeated Von Frey (last 5s)	5.49	3.54	6.955	41	< 0.001	1.451
Heat	3.34	2.45	3.887	34	0.001	0.878

Note. Reported *p*-values were adjusted for multiple comparisons using the Benjamini-Hochberg method

### Group differences

**Fig. 2:**
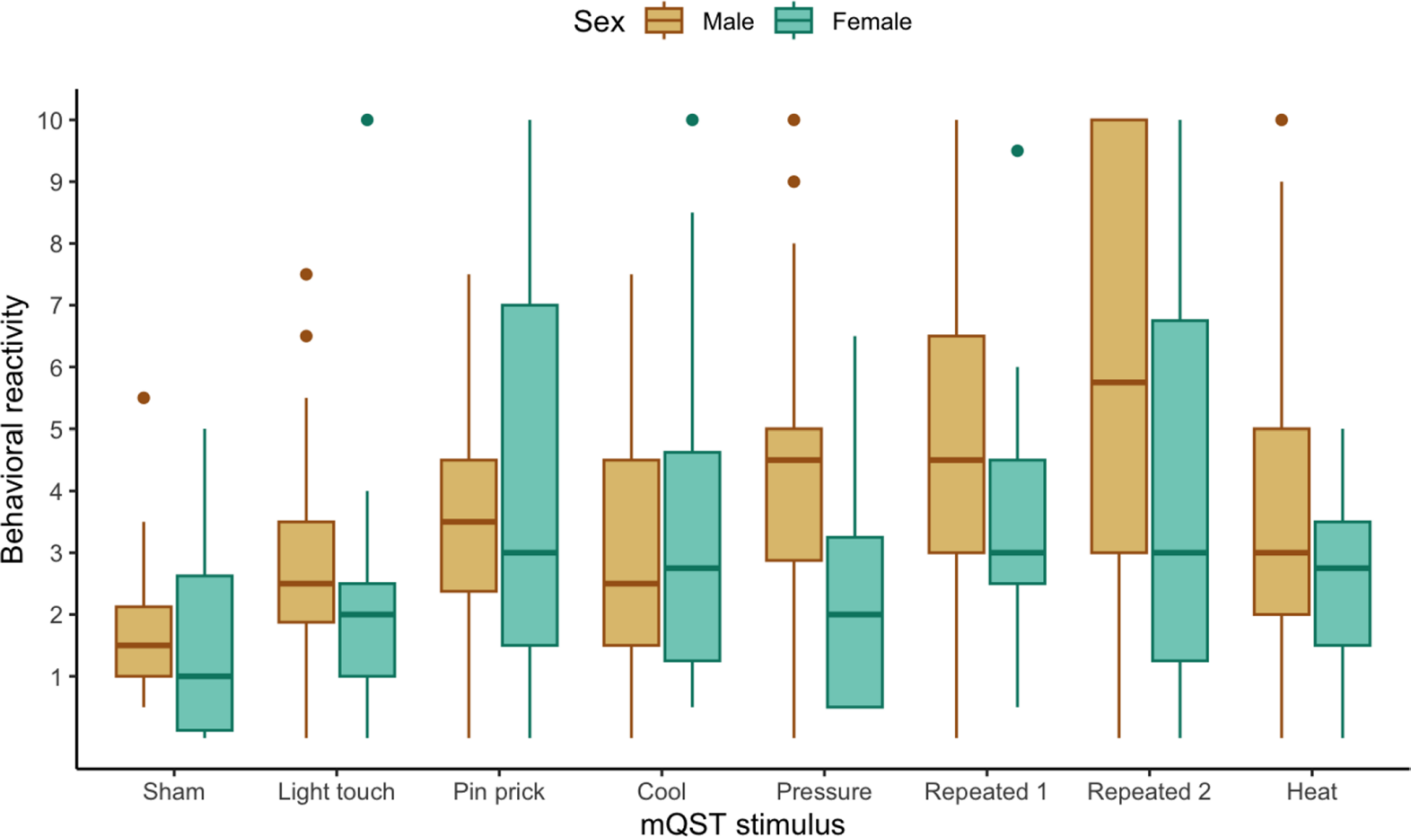
Boxplot Displaying Behavioral Reactivity Across mQST Stimuli for Male (*n* = 33) and Female (*n* = 14) Participants. *Note.* Sex represents participants assigned male or female at birth. Repeated 1 indicates behavioral reactivity during the first 5 s and Repeated 2 indicates behavioral reactivity during the last 5 s of the 30-second von Frey application

**Fig. 3:**
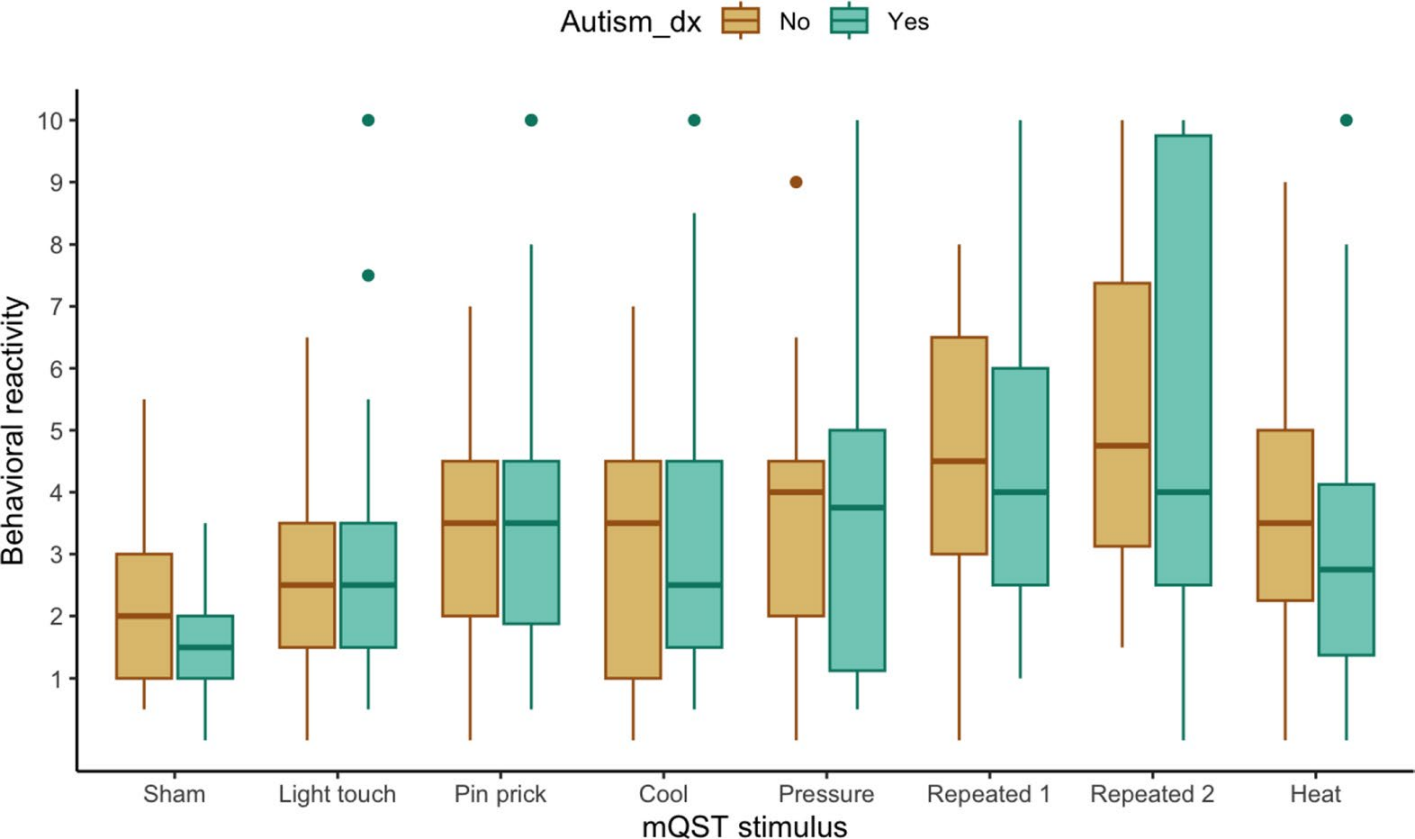
Boxplot Displaying Behavioral Reactivity Across mQST Stimuli for Participants with (*n* = 34) and without (*n* = 13)ASD. Note. The Autism_dx group “no” represents participants with developmental delay/disability without an ASD diagnosis, and “yes” represents participants with an autism diagnosis. Repeated 1 indicates behavioral reactivity during the first 5 s, and Repeated 2 indicates behavioral reactivity during the last 5 s of the 30-second von Frey application

**Fig. 4:**
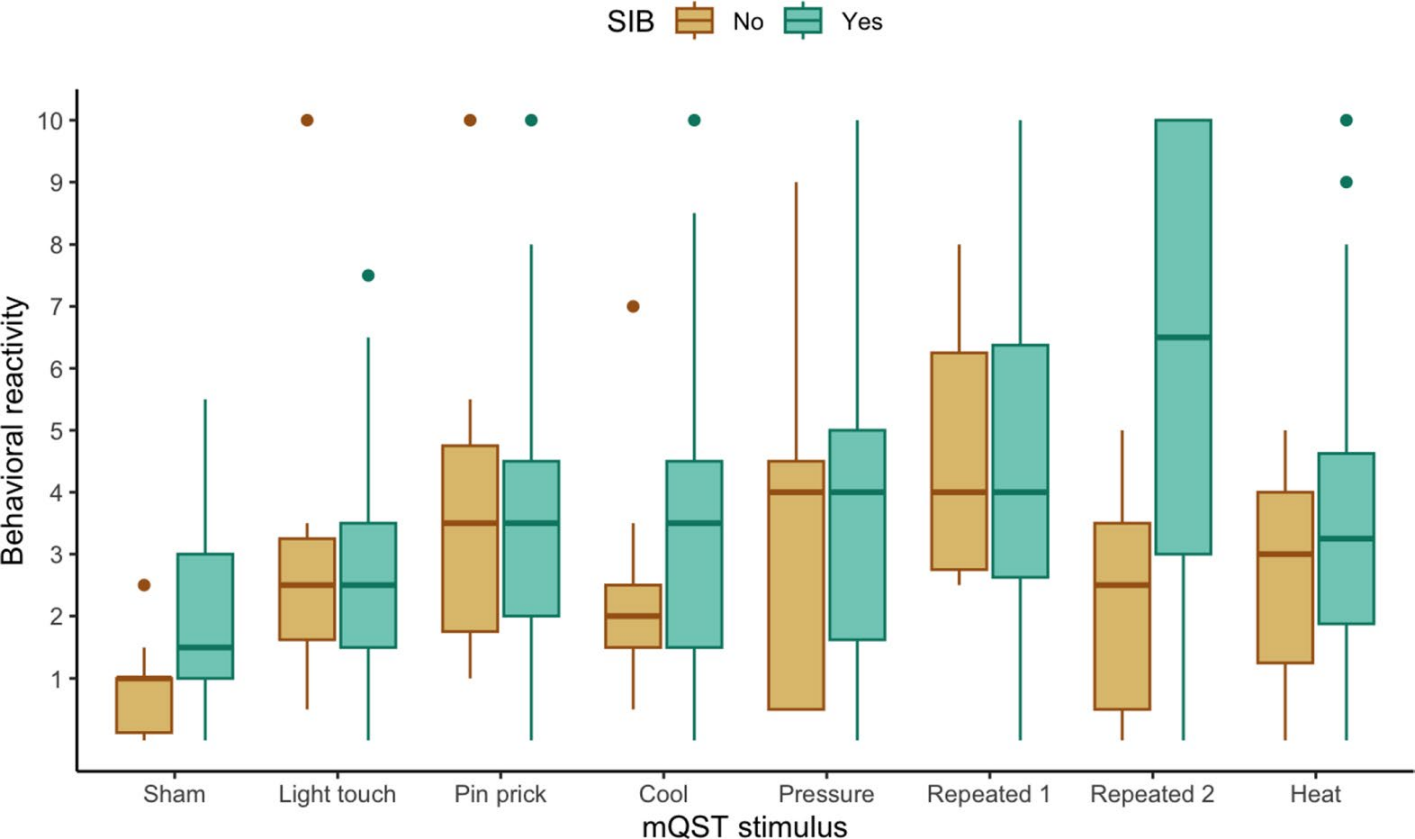
Boxplot Displaying Behavioral Reactivity Across mQST Stimuli for Participants with (*n* = 36) and without (*n* = 11) SIB. *Note.* The no-SIB group represents participants with 0 endorsed self-injurious behaviors on the RBS-EC. The SIB group represents participants with 1 or more endorsed SIB items on the RBS-EC

We explored if behavioral reactivity to mQST stimuli differed by demographic, diagnostic, or SIB variables. Based on preliminary visual analysis depicted in box and whisker plots, behavioral reactivity did not differ by sex (see Fig. 2) or diagnostic subgroup (ASD; see Fig. 3). The preliminary visual analysis of the box and whisker plots comparing participants with or without endorsed SIB suggests a potential group difference in behavioral reactivity to the repeated Von Frey last 5s of application (see Fig. 4).

**Fig. 5:**
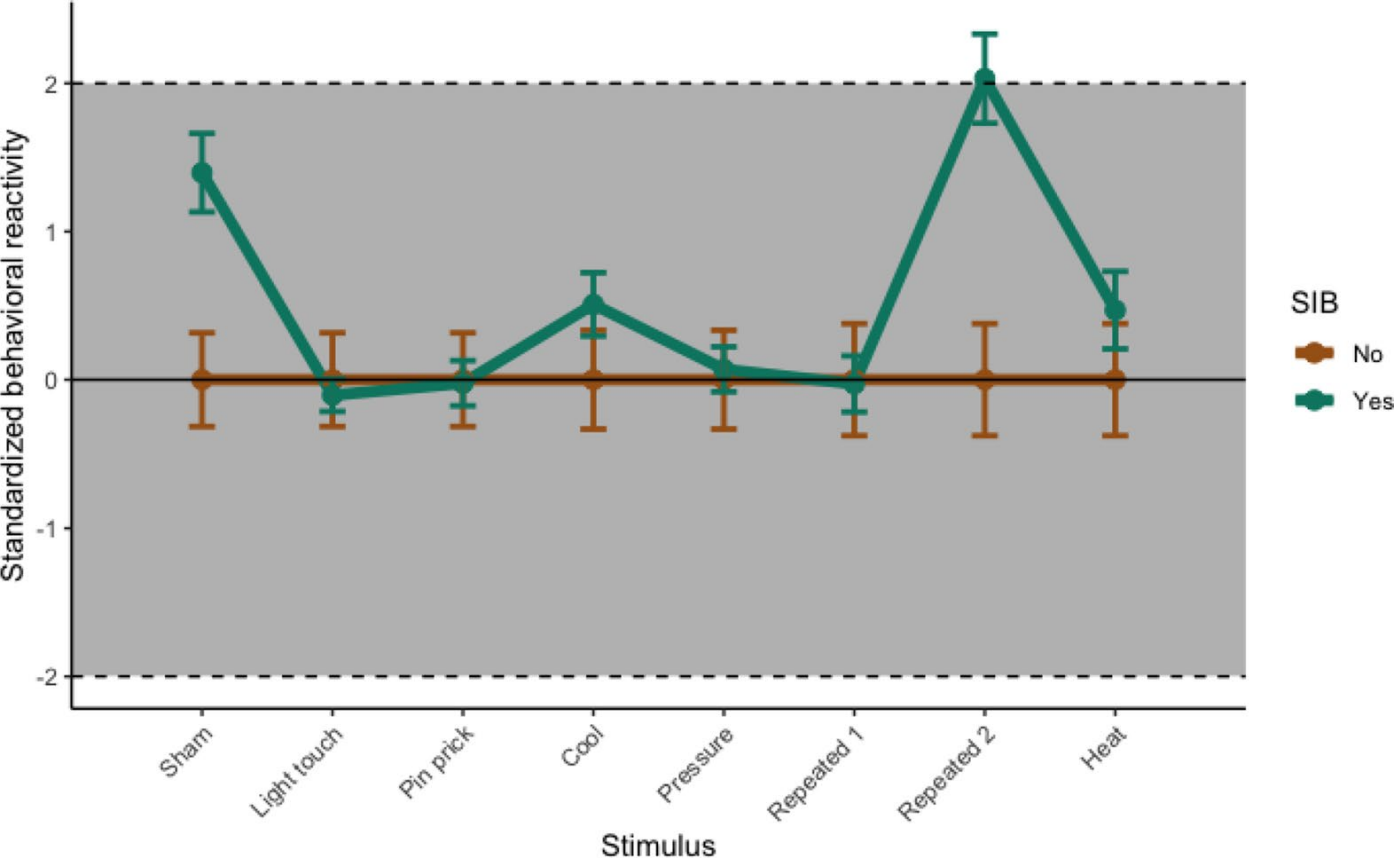
Average Standardized Behavioral Reactivity Scores for Participants with and without SIB. *Note.* The y-axis displays standard deviations for standardized behavioral reactivity scores. Behavioral reactivity scores were *z*-transformed against the group of participants without SIB. Average group score (and standard error) is plotted for each stimulus for individuals who do (green line) and do not (brown line) endorse SIB. The horizontal dashed lines indicate two standard deviations away from the mean for the group of participants without SIB. The gray confidence envelope defines the area encompassing two standard deviations above and below the mean

The potential group difference was further investigated using two-sample *t*-tests. As a subgroup, children reported to engage in SIB showed significantly more behavioral reactivity to the repeated von Frey last five seconds (*M* = 6.14, SD = 3.44), *t* (40) = − 2.247, *p* =.047) compared to children without SIB (see Table 3) after adjusting for multiple comparisons. Behavioral reactivity to other sensory stimuli (light touch, pin, cool, pressure, heat) was comparable across children who did and did not engage in SIB (See Fig. 4). Figure 5 displays behavioral reactivity scores z-transformed against the group of participants without SIB. Mean reactivity for the SIB group during the last 5 s of the repeated von Frey was outside two standard deviations above the mean reactivity for the no SIB group.

**Table 3:** Two-sample t-tests examining differences in behavioral reactivity to mQST stimuli between participants who Self-Injure compared to Non-Self-Injuring participants (RBS-EC)

mQST Stimulus	No SIB Group (*n* = 10)		SIB Group (*n* = 36)		t	df	*p*	Cohen’s d
	*M*	*SD*	*M*	*SD*				
Sham	0.85	0.78	1.94	1.25	-2.624	44	0.083	**-0.938**
Light touch	2.95	2.66	2.67	1.73	0.396	43	0.950	0.142
Pin	3.75	2.71	3.69	2.45	0.072	43	0.950	0.026
Cool	2.39	1.93	3.37	2.44	-1.116	42	0.950	-0.417
Pressure	3.56	2.78	3.75	2.46	-0.206	41	0.950	-0.077
Repeated Von Frey (first 5s)	4.64	2.41	4.57	2.67	0.064	39	0.950	0.026
Repeated Von Frey (last 5s)	2.21	1.93	6.14	3.44	-2.910	40	**0.047**	**-1.206**
Heat	2.64	1.86	3.52	2.58	-0.840	33	0.950	-0.355

Note. The no-SIB group includes participants with a score of 0 on the self-directed behaviors subscale on the RBS-EC. The SIB group includes participants with a score of 1 or more on the self-directed behaviors subscale on the RBS-EC. Reported *p*-values were adjusted for multiple comparisons using the Benjamini-Hochberg method

Last, there was no statistically significant association between SIB status and test termination criteria (two-tailed *p* =.384). Nine participants terminated the mQST test due to signs of distress across two applications in a row or the expression of tears. Of the participants that met criteria for termination of the mQST protocol, 5 (55%) were male, ranged in age from 3 to 10 years, 6 (66%) endorsed SIB on the RBS-EC (*M* = 4.33, *SD* = 6.04, range = [0, 16]), 8 (89%) were diagnosed with ASD.

## Discussion

We found that the mQST protocol was feasible and reliably coded in a sample of children with NDDs of varying severity and etiology. There was significantly greater behavioral reactivity associated with active tactile stimuli versus sham (no active), providing validity evidence that the mQST protocol is capturing behavioral reactivity through blinded scoring across vocal, facial, and gross motor responses. We further examined whether behavioral reactivity differed by demographic, clinical, or SIB variables. On average, the children with caregiver-reported SIB showed significantly greater reactivity to the last 5 s of the 30-second application of the repeated von Frey and similar reactivity to other stimuli as compared to children without SIB. These results add to the current knowledge regarding associations between pain/tactile reactivity and SIB among children with NDDs, providing further evidence that pain insensitivity was not associated with SIB in this sample.

The current feasibility results add to previous research showing that the mQST can be applied with reliable behavioral coding in populations with complex communication needs [17–19, 32, 33]. In the current study, 91% of behavioral reactivity scores across stimulus types could be calculated, leaving 9% missing due to either technical issues (visibility of the face or body) or not applying the stimuli (termination of the mQST test). Using the manualized behavior coding criteria, independent coders showed strong inter-observer agreement, on average (84.7%). These results build on previous research supporting the notion that non-verbal behavior among individuals with NDDs can be reliably quantified and associated with painful events [14, 15, 41, 42]. While the mQST stimuli are likely all non-noxious (although may vary on degrees of discomfort, i.e., pin, heat), the current results show that facial, verbal, and motor behaviors correspond to the calibrated application of tactile stimuli implicated in afferent sensory circuits important for transduction (presumably A beta [Aβ] and delta [Aδ] fibers) [43]. In the current study, behavioral reactivity was significantly elevated during the active application of stimuli as compared to the sham (no active touch), adding evidence that the mQST protocol can be used to measure tactile sensory reactivity. Similarly, Symons et al. [17] applied the mQST protocol with adults with ID and found greater behavioral reactivity during stimuli application versus a sham application.

Concerning SIB, the current results suggest that the children with SIB showed as much and, in some cases (e.g., repeated von Frey), more tactile behavioral reactivity compared to children without SIB. These results are important considering a hypothesized role of pain insensitivity involved in some models of SIB. The opioid model of SIB implicates endogenous opioid and neuroendocrine mechanisms involving the hypothalamic-pituitary-adrenal system (HPA axis), specifically, the proopiomelanocortin molecule from which beta-endorphin is cleaved, which is dysregulated among at least some SIB subgroups [44]. This pain perspective holds that, due to elevated opioid levels, individuals with SIB have an increased pain tolerance and engage in the behavior without pain (i.e., there is no natural aversive consequence or ‘brake’). Much of the work on dysregulated opioid systems and SIB is from adult samples with severe SIB. In our pediatric sample for which SIB is present but with far shorter chronicity, children were not less sensitive to potentially noxious stimuli (pin, heat). Similarly, Breau et al. [45] examined behavioral pain reactivity in a sample of 44 children with SIB and 57 without, reporting that pain behavior did not differ significantly across SIB status and that pain reactions were comparable across groups during an observed pain episode. If not a developmental factor, there could be a chronicity or severity factor to account for the adult and pediatric differences around SIB and pain reactivity; although speculative, there may be an interaction between the length of time tissue damaging SIB is part of the behavioral repertoire, particularly if it is not effectively treated, and engagement with neuroendocrine and stress regulatory pathways, particularly opioid-mediated, leading to altered sensory thresholds for tactile and/or noxious stimuli. For adults with severe SIB and ID, there is clear evidence that there are responders to opiate antagonists, further implicating endogenous opioid systems and SIB [46, 47]. Alternatively, perhaps sensory (tactile/nociceptive) receptive fields are influenced by the degree and location of tissue damage for some forms of SIB over different time scales. In an earlier work, Symons and Thomspon found correlative evidence that some SIB body sites in a sample of adolescents with ID corresponded to known (empirically verified) acupuncture (mechanical or electrical) analgesia sites, presumably mediated by endogenous opioids [48], 

Differences in behavioral reactivity to the last 5 s of repeated von Frey application may indicate differences in summation or a wind-up effect between individuals who self-injure compared to those that do not. A wind-up effect refers to a progressive increase in action potential frequency with repetitive firing of peripheral nociceptors. This result may be evidence of hyperalgesia consistent with central sensitization. There are a fair number of case studies linking SIB to painful health conditions (e.g., GERD, ear infections), suggesting, for at least some children, SIB may be a response to an underlying chronic pain condition contributing to central sensitization [49, 50]. On the other hand, it has been hypothesized that SIB would be associated with decreased wind-up/sensitization based on the logic that more sensitization would serve as a punisher for SIB [51]. It is possible that our results are not specific to temporal summation, as the QST protocol most closely aligned with a test of wind up uses a repeated von Frey vs. repeated pin prick (which would be difficult to obtain ethical approval in a vulnerable population).

In relation to sex as a biological variable, our preliminary analysis with a relatively small sample of females (*n* = 14) did not find behavioral reactivity differences by sex. In an adult sample with more fine-grained behavioral coding (facial action coding), Symons et al. [17] found elevated facial responses in females compared to males broadly across the mQST. It is prudent that future work should continue to consider potential sex differences in sensory expression across facial, vocal, and motor behaviors among individuals with intellectual disability and associated neurodevelopmental conditions.

Sensory symptoms commonly co-occur in ASD (up to 93%) [52] and are found to differentiate ASD from other developmental disorders [53]. Studies aimed to characterize abnormal responsivity report variable and overlapping symptoms commonly described as under-responsive, over-responsive, and failure to habituate [54]. This body of literature, however, is dominated by caregiver or self-report measures that provide subjective information related to a person’s affective reactions [55]. Yet, the limited number of studies that used psychophysical assessments report similar findings to those in the current sample, that tactile differences are not found in relation to an ASD diagnosis. For example, one study with 13 adults diagnosed with ASD and 13 matched non-autistic adults with IQ in the normal range found no group differences between somatosensory detection and pain thresholds [56]. In another study using QST approaches, the individuals with ASD had higher thresholds for mechanical pain compared to the control group, however data for both groups remained within the normal distribution of healthy individuals, suggesting that the reported difference is not clinically significant [57]. It is possible that ASD somatosensory differences lie in more specific mechanoreceptors (e.g., pressure, vibration, skin distortion). Flutter thresholds (vibrotactile stimuli < 50 Hz), for example, are reported to be reduced in samples with ASD, suggestive of altered inhibition [58, 59]. This line of research requires more studies with larger sample sizes to make meaningful inferences.

It is important to point out the limitations of the study. The sample was not randomly selected so the results are specific to the sample. Any consideration of the generality of findings is based on logical generalization only and should be done with caution. The results should also be considered tentative given the relatively small and heterogeneous sample of children and exploratory methods investigating preliminary group differences without controlling for confounding variables such as age, sex, or diagnostic category. While less than 10% of mQST stimuli scores were missing, it is important to note that the current analysis did not evaluate the impact of missingness on group differences. The individual variability observed in reactivity to tactile stimuli should be further explored. Future studies should examine larger samples, permitting a more robust investigation of the relationship between co-occurring diagnoses and level of intellectual functioning, with respect to tactile reactivity. SIB was measured from parent report only and not corroborated/confirmed by direct observation; nor were we able to consider occurrence of SIB in relation to behavioral reactivity (e.g., so-called ‘good day/bad day’ in terms of a day with high rates of SIB vs. a day with limited SIB and the relation therein with behavioral reactivity). The SIB group in our sample represented children whose parents observed SIB to varying degrees from interfering very little with daily living to always interfering. Future research should also examine the degree to which the results of the mQST results correlate with caregiver-reported sensory symptoms, which were not collected in this study.

Overall, a modified QST protocol was successfully implemented with a heterogenous pediatric sample with NDDs in a clinical context and with reliable behavioral measurement. The study provides further evidence establishing mQST as a feasible clinical research tool in this setting. Substantively, as the scientific understanding of sensory variables in relation to early development and behavioral function in children with or at risk for NDDs continues to grow, consideration of a more direct, quantitative approach to eliciting and evaluating behavioral reactivity to calibrated tactile stimuli may be a useful complement to current indirect measures based on questionnaires and rating scales.

## Data Availability

Data generated for this article are available from the corresponding author on reasonable request.
